# iPSC-neural crest derived cells embedded in 3D printable bio-ink promote cranial bone defect repair

**DOI:** 10.1038/s41598-022-22502-8

**Published:** 2022-11-04

**Authors:** Juliane D. Glaeser, Xianchao Bao, Giselle Kaneda, Pablo Avalos, Phillip Behrens, Khosrowdad Salehi, Xiaoyu Da, Angel Chen, Chloe Castaneda, Pawel Nakielski, Wensen Jiang, Wafa Tawackoli, Dmitriy Sheyn

**Affiliations:** 1 Orthopaedic Stem Cell Research Laboratory, Cedars-Sinai Medical Center, 127 S San Vicente Blvd. AHSP A8308, Los Angeles, CA 90048 USA; 2 Board of Governors Regenerative Medicine Institute, Cedars-Sinai Medical Center, 127 S San Vicente Blvd. AHSP A8308, Los Angeles, CA 90048 USA; 3 Department of Orthopaedics, Cedars-Sinai Medical Center, 8700 Beverly Blvd. AHSP A8308, Los Angeles, CA 90048 USA; 4 Department of Surgery, Cedars-Sinai Medical Center, 8700 Beverly Blvd. AHSP A8308, Los Angeles, CA 90048 USA; 5 Cedars-Sinai Medical Center, Biomedical Imaging Research Institute, 116 N Robertson Blvs. Pacific Theatres Building, Suite 400, Los Angeles, CA 90048 USA; 6 Department of Biomedical Sciences, Cedars-Sinai Medical Center, 8700 Beverly Blvd. AHSP A8308, Los Angeles, CA 90048 USA; 7 Department of Orthopedics, West China Hospital, Sichuan University, Chengdu, China; 8 Department of Biosystems and Soft Matter, Institute of Fundamental Technological Research Polish Academy of Sciences, 02-106 Warsaw, Poland

**Keywords:** Induced pluripotent stem cells, Stem-cell research, Translational research

## Abstract

Cranial bone loss presents a major clinical challenge and new regenerative approaches to address craniofacial reconstruction are in great demand. Induced pluripotent stem cell (iPSC) differentiation is a powerful tool to generate mesenchymal stromal cells (MSCs). Prior research demonstrated the potential of bone marrow-derived MSCs (BM-MSCs) and iPSC-derived mesenchymal progenitor cells via the neural crest (NCC-MPCs) or mesodermal lineages (iMSCs) to be promising cell source for bone regeneration. Overexpression of human recombinant bone morphogenetic protein (BMP)6 efficiently stimulates bone formation. The study aimed to evaluate the potential of iPSC-derived cells via neural crest or mesoderm overexpressing BMP6 and embedded in 3D printable bio-ink to generate viable bone graft alternatives for cranial reconstruction. Cell viability, osteogenic potential of cells, and bio-ink (Ink-Bone or GelXa) combinations were investigated in vitro using bioluminescent imaging. The osteogenic potential of bio-ink-cell constructs were evaluated in osteogenic media or nucleofected with BMP6 using qRT-PCR and in vitro μCT. For in vivo testing, two 2 mm circular defects were created in the frontal and parietal bones of NOD/SCID mice and treated with Ink-Bone, Ink-Bone + BM-MSC-BMP6, Ink-Bone + iMSC-BMP6, Ink-Bone + iNCC-MPC-BMP6, or left untreated. For follow-up, µCT was performed at weeks 0, 4, and 8 weeks. At the time of sacrifice (week 8), histological and immunofluorescent analyses were performed. Both bio-inks supported cell survival and promoted osteogenic differentiation of iNCC-MPCs and BM-MSCs in vitro. At 4 weeks, cell viability of both BM-MSCs and iNCC-MPCs were increased in Ink-Bone compared to GelXA. The combination of Ink-Bone with iNCC-MPC-BMP6 resulted in an increased bone volume in the frontal bone compared to the other groups at 4 weeks post-surgery. At 8 weeks, both iNCC-MPC-BMP6 and iMSC-MSC-BMP6 resulted in an increased bone volume and partial bone bridging between the implant and host bone compared to the other groups. The results of this study show the potential of NCC-MPC-incorporated bio-ink to regenerate frontal cranial defects. Therefore, this bio-ink-cell combination should be further investigated for its therapeutic potential in large animal models with larger cranial defects, allowing for 3D printing of the cell-incorporated material.

## Introduction

In cases of extreme bone loss following a traumatic injury, in which the mechanism of bone self-repair is inadequate, grafts or alloplastic materials are typically used to repair the defect. The current market value for craniofacial bone replacement is estimated to be $390 million for trauma alone, representing 13% of all traumatic bone injuries^1^. The “gold standard” for stimulating new bone formation is autologous bone grafting^2^. However, donor-site morbidity is a limiting factor^3^. Cranioplasty with alloplastic materials like Titanium or polymers like polymethyl methacrylate and polyether ether ketone are often associated with high rates of infection and complications^3^. The introduction of three-dimensional computed tomography has revolutionized calvarial reconstruction through the creation of anatomic alloplastic models^4^. However, these models typically need to be combined with autografts. Furthermore, alloplastic materials are not ideal for use in children and juveniles due to cranial growth. Recombinant osteogenic growth factors, such as recombinant human bone morphogenetic proteins (rhBMP), are only used to treat small bone lesions due to their high cost and safety concerns including inflammation and swelling^1,5^.

3D bioprinting is a developing technology that can create scaffolds from different biomaterials that precisely mimic the shape, size, and dimensions of a defect^6^. Although many 3D bioprinting methods, including those used in dentistry, produce constructs with suitable interconnected porosity and mechanical properties, they often require the application of high temperatures, solvents, or other conditions that are incompatible with living cells^7^. Post-production seeding of cells has been reported to result in non-uniform cell distribution and poor cell attachment^8^. A potential solution to include cells in 3D printing is the use of soft bio-inks that are crosslinked post-production. These bio-inks include synthetic or natural polymers^10–11^. An ideal bio-ink is printable into different structures, mechanically stable, cytocompatible, non-immunogenic, and has a suitable degradation rate^12^. One option is to use alginate, an inexpensive biomaterial that has been widely studied for diverse biomedical applications including cartilage and bone^10,13^, due to its excellent cell responses and flexible gelation preparation through divalent ions including calcium. An interesting material was recently reported by Lu et al.^14^. An instantly fixable and self-adaptive scaffold by dopamine-modified hyaluronic acid chelating Ca^2+^ of themicrohydroxyapatite surface and bonding type I collagen that highly simulates the natural bony matrix, which promoted cranial regeneration by autologous stem cell recruitment and angiogenesis in small and large animal models in a recent study^14^.

Bone marrow-derived Mesenchymal Stromal Cells (BM-MSCs) are the most utilized cells for long bone regeneration due to their accessibility^15–18^. In the challenging environment of cranial bone, repair of large defects was achieved by BMP-2 expressing bone marrow stromal cells^19^. Craniofacial bones are flat, formed through intramembranous ossification, and develop from embryological origins distinct from those of long bones^20^. Within the cranium, only the parietal bones are of mesodermal origin, whereas the formation of frontal cartilage and bone originates in the neural crest (NC)^21,22^. Interestingly, a higher regeneration potential has been shown in NC-derived compared to mesoderm-derived calvarial bones^23^. Furthermore, cells obtained from NC bone were shown to be more osteogenic, more proliferative, and less apoptotic^24–26^. During embryogenesis, neural crest cells (NCCs) are identified within the dorsal margins of the closing neural fold^27^, then migrate into various skeletal tissues^28^. Their profound role in cranial skeletogenesis makes NCCs an attractive cell source for cranial repair. Although this cell type is rare in adults, it can be obtained through the differentiation of induced pluripotent stem cell (iPSC)^29–31^. Our prior study demonstrates that iPSC-derived NC mesenchymal progenitor cells (iNCC-MPC) are more efficient in revitalizing cranial allografts than BM-MSCs^32^. Since cell source and mode of differentiation both may affect the cells’ performance, a direct comparison between iNCC-MPCs and iMSCs should be performed^32^.

The BMP family and its 20 identified members play an important role in osteogenesis^33,34^. Adenovirus-mediated MSC transduction with 14 different human isoforms of BMP revealed that BMP2, BMP6, and BMP9 are the most potent inducers of osteogenesis in MSCs^35,36^. Although less popular than BMP2, BMP6 overexpression in BM-MSCs or adipose-derived stromal cells was demonstrated to be more potent in term of stimulation of bone formation^37^.

In prior research, we demonstrated that implantation of iNCC-MPC-seeded allografts results in an increased revitalization of cranial bone grafts compared to BM-MSCs^32^ and that BMP6 overexpression of in MSCs efficiently stimulates bone regeneration^37–41^. BMP6 overexpressing iNCC-MPCs in combination with the use of bioprintible bio-ink might be a promising alternative to the use of iNCC-MPC-seeded allografts. Due to the neural crest origin of frontal bone, this combinatory cell therapy approach may be more efficient in the frontal bone area of the cranium. As highlighted in the review of Soman and Vijayavenkataraman, iPSC technology and advancements in 3D bioprinting technology may enable 3D printing of iPSC-derived constructs with native tissue architecture and function^42^.

## Methods

### Experimental design

**Figure 1:**
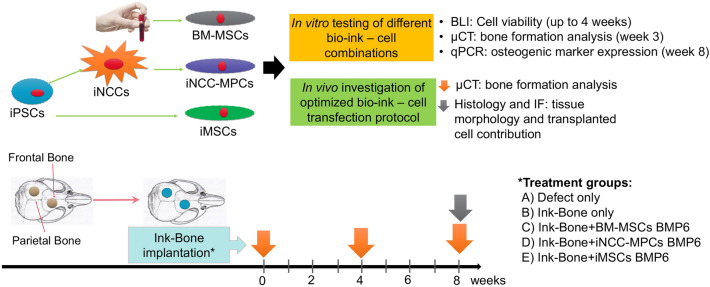
Graphical abstract and experimental design.

Two types of bio-inks, CELLINK BONE (hereafter referred to in short Ink-Bone) or GelXA BONE (in short GelXA, both CELLINK Life Sciences, Boston, MA), were investigated in this study. For in vitro analysis, the cell viability and osteogenic potential of BM-MSCs and iNCC-MPCs were investigated when combined with the bio-inks. For cell viability testing, bioluminescent imaging (BLI) was performed for a duration of 4 weeks. For cell visualization, cells were transfected with a luciferase reporter gene prior to testing. For testing of the osteogenic differentiation potential, cells, combined with each of the bio-ink types, were exposed to either osteogenic media for a duration of 8 weeks or nucleofected with rhBMP6 (or GFP control) prior to bio-ink exposure. At week 8, RNA was isolated from the cell bio-ink constructs and qPCR analysis was conducted using osteogenic primers. For in vivo testing, two 2 mm circular defects were created in the frontal and parietal bone of NOD/SCID mice (*n* = 39). According to the literature these defects are critical-sized (at least 1.8 mm in diameter)^43^. Each defect was treated as follows: (A) untreated (*n* = 5), (B) with Ink-bone only (*n* = 8), (C) with Ink-Bone + BM-MSC-BMP6 (*n* = 5), (D) with Ink-Bone + iMSC-BMP6 (*n* = 7), or (E) with Ink-Bone + iNCC-MPC-BMP6 (*n* = 14). For follow-up, µCT was performed at weeks 0, 4, and 8 weeks. At the time of sacrifice (week 8), histological and immunofluorescent analyses were performed (Fig. 1). All the methods were performed in accordance with relevant guidelines and regulations.

### Cell derivation, culture, and luciferase transduction

Human BM-MSCs were isolated from whole bone marrow aspirate (Lonza, USA), using the standard method and plastic adherence, as previously described^41,44–48^. Induced MSCs were differentiated from iPSCs, as previously published by our group^41^. Neural crest cells were differentiated from iPSCs and then further differentiated to iNCC-MPCs, as previously described^30,31^. For investigation of cell viability in vitro, BM-MSCs and iNCC-MPCs were transduced with a lentiviral vector harboring the reporter gene luciferase2 (Luc2) under the constitutive ubiquitin promoter to allow in vivo imaging of cell survival, as previously reported^39,49^. The overexpression of the reporter gene *green fluorescent protein (GFP)* or the osteogenic gene *BMP6* was achieved using the pCMV-EGFP-N1 and pCMV-cDNA3-rhBMP-6 plasmids respectively with the aid of the Nucleofector™ device (Lonza) and a MSC-specific nucleofection buffer, as we previously reported^37–39,41,50^. Immediately after nucleofection, the cells were plated in a complete growth medium containing 20% FBS and were maintained in culture for 24 h before 3D printing or implantation.

### Preparation of cell-seeded bio-inks, 3D printing, and scanning electron microscopy

Two types of bio-inks, Ink-Bone or GelXA were combined with either BM-MSCs or NCC-MPCs derived from induced pluripotent stem cells (iNCC-MPCs) in a 9:1 ratio (bio-ink:cells). Briefly, cells were lifted with 0.25% trypsin after washing with PBS, counted, and stained with DiI (Invitrogen, Carlsbad, CA) in PBS for 20 min. Cells were washed twice with PBS and pelleted. 900 μl of CELLINK BONE or GelXA was combined with 10 million either BM-MSC-BMP6, iNCC-MPC-BMP6, or iMSC-BMP6 cells resuspended in 100 µl 1xPBS (GIBCO, Waltham, MA), to achieve 1:9 cell to bio-ink ratio. The bone-graft/cell combination was then transferred to a 3 ml syringe. A luer lock adaptor was used to connect another 3 ml syringe and mix the two syringes until the cell suspension was homogeneous.

The 3D printing was performed using a Cellink Bio X™ 3D bioprinter. The constructing design is a 10 mm × 10 mm × 2 mm square. Cells were pre-cultured to confluency and pre-mixed with the cellink bone using the recommended protocols from Cellink. The constructs were printed at 25–30 kpa at 9 m/ml concentration. The printhead was a 22-G plastic needle and the print speed was 10 mm/s. For samples used for SEM imaging, a 25-G plastic needle was used. The constructs were printed into a Petri dish. The Petri dish was then removed from the printer, and a Ca^2+^ crosslinking agent from Cellink was used to crosslink and solidify the construct.

The printed construct was frozen in liquid nitrogen before cross-sectional cutting and freeze-drying. Before imaging, samples were sputtered with gold using an SC7620 Polaron mini sputter coater (Quorum Technologies Ltd., Ashford, UK). Filament morphology and cross-section were assessed with the scanning electron microscope (SEM JEOL JSM-6390LV, JEOL, Tokyo, Japan).

### Bioluminescence imaging

To measure the viability of the cells seeded on the bio-inks, Luc2 expression was quantified using Bioluminescence Imaging (BLI) at days 1, 4, 7, 10, 14, 21, and 28, as described previously^39,49,51^. The IVIS camera image capture was set starting from 8 to 10 min after D-luciferin injection for peak BLI with a target max count (3000 or 30,000 photon counts) and auto-exposure time (with a maximum exposure time of 1 min) were set before acquiring the images. A circular region of interest (ROI, 7-mm in diameter) was applied to all acquired images to measure the total flux (photons per sec) in the ROI. Image acquisition and analysis were performed with Living Image software packages (Version 4.5.5). The image analysis was done using total influx data calculated from the same size ROI (one well), normalized to the background noise of each image. BLI signals were normalized to signals detected on day 1.

### Cell nucleofection with BMP6

For cell nucleofection, the Amaxa Human MSC Nucleofector Kit was used (Lonza, Basel, Switzerland). Cells were washed with PBS (GIBCO) then lifted with 0.25% Trypsin–EDTA (Sigma, St Louis, MO) and counted and aliquoted at 1 × 10^6^ cells per 15 ml conical tube. 2 mL of DMEM (GIBCO) with 20% FBS was placed in 6-well plates and pre-warmed in a 37 °C incubator. Cells were resuspended in 100 μl nucleofection solution (Lonza) and 10 μg of BMP-6 plasmid was added to the solution and placed in a cuvette. The cuvette was placed in the Nucleofector II device (Lonza) and set to program G-22. After nucleofection, cells were immediately pipetted out of the cuvette and into the 6-well plate with pre-warmed media. The final concentration in each well was 2 × 10^6^ cells. The next morning the cells were lifted as described above, counted, and used for the preparation of cell-seeded bio-inks.

### Differentiation and qPCR testing

For testing of the osteogenic differentiation potential, both bio-inks were either combined with BMP6 (GFP control) nucleofected cells or non-transfected cells that were then exposed to osteogenic media for a duration of 8 weeks (or GFP control). The osteogenic media contained 0.1 µM Dexamethasone, 10 mM β-glycophosphate, 50 µg/ml L-Ascorbic acid, as previously described^32,38,52,53^. The cells’ differentiation potential was confirmed at week 8 by osteogenic gene marker expression. RNA was isolated from the cell bio-ink constructs, transcribed into cDNA, and qPCR analysis conducted, as previously described^32,37,52,54,55^. Taqman Gene Expression Assay for: osteocalcin (Oc), collagen type I (Col I), alkaline phosphatase (ALP), and osteonectin (On) were used for analysis.

### Calvarial surgery

Animal surgeries were performed in accordance with the approved Cedars-Sinai Institutional Animal Care and use Committee (IACUC) protocol #007961, as previously reported^56^. All animal procedures were performed according to the ARRIVE guidelines^57^. To create calvarial defects, the 6–8-week-old NOD/SCID mice (NOD.CB17-Prkdcscid/NCrHsd, Envigo, Indianapolis, IN) were anesthetized with 2–3% isoflurane, and buprenorphine (0.05 mg/kg) was injected subcutaneously (SQ) for analgesia. Animals were given thermal support for the duration of the procedure. The surgical site was shaved and aseptically prepped by thoroughly disinfecting with betadine and 70% isopropyl alcohol. Animals were placed on a stereotaxic instrument, with the skull secured with ear bars and a tooth bar. A straight incision (~ 15 mm) was made over the midline to expose the parietal and frontal bones. Two 2 mm-diameter full-thickness circular skull defects, one in the parietal bone (alternating left and right of the midline suture) and one in the frontal bone (alternating left and right of the midline suture, opposite side to the parietal bone defect), were made using a Dremel drill. The drilling angle from the midline suture was between 1-2 mm to the left and right, respectively. Each defect was treated according to the experimental design. For defect filling, 10 μl of Ink-Bone suspension with or without cells (measured with a 250 μl Hamilton) was added per defect area via wax spatula followed by the addition of a single drop of Crosslinking Agent (Cellink Life Sciences) for 30 s. Afterward, the agent was carefully removed with sterile gauze.

The skin incision was sutured using monofilament nylon non-absorbable suture (5–0 Ethilon black) in a horizontal mattress pattern. The animals were then removed from the stereotaxic instrument and given 1 ml of 0.9% saline SQ. Animals were observed and buprenorphine (0.05 mg/kg) was administered subcutaneously early in the morning on the day after surgery and as needed thereafter. The antibiotic enrofloxacin was administered daily for 5 consecutive days (5 mg/kg; 0.06 ml; SQ injection) to prevent infection. Sutures were removed 7–10 days after the completion of the surgery. Mice were housed in groups of 5 per cage for the whole study duration.

### In vivo µCT analysis

Bone formation was monitored by µCT analysis in vivo, as previously described^39,58,59^. The mice were scanned post-surgery using a Viva CT 40 (Scanco Medical, Wangen-Brüttisellen, Switzerland) at day 1, week 4, and 8. Bone volume (BV), bone mineral density, and connectivity density^60^ were evaluated, as previously described^39,58^.

The mice were placed inside an induction chamber and anesthetized using a 4% isoflurane-oxygen mixture for approximately 2 min. The animals were transferred from the induction chamber to the 34.8-mm sample holder where anesthesia was maintained with 1.5–2% isoflurane delivered via a nose cone. Special care was taken to position the mice on their abdomen. The scanner was set to have a field of view of 20.5 mm, X-ray energy of 55 kVp, the intensity of 145μA, using 1,000 projections per 360°, the integration time of 200 ms, and reconstructed at a spatial nominal resolution of 35 µm. The defect margins were aligned to a standard position, and a cylindrical volume of interest was defined (1.58 mm in diameter, including partial host bone in the outer periphery, and an average of 35 slices in depth). A constrained 3D Gaussian filter was used to partially suppress noise in the volumes. The bone tissue was segmented by using a global thresholding procedure. Week 4 and 8 data were normalized to day 1 data obtained from the same animal to reduce variation.

### Histological and immunofluorescent analyses

After sacrifice at week 8 post-surgery, the defect site including the allograft and surrounding bone tissue was explanted. Samples were fixed in in 4% formaldehyde solution, decalcified by incubation in 0.5 M EDTA (pH 7.4) for 3 weeks, passed through a graded series of ethanol solutions, and embedded in paraffin. Five-micron-thick sections were cut from the paraffin blocks. Hematoxylin and eosin (H&E) staining was performed to evaluate the morphological features of the healing process, graft-to-host osseointegration, and fibrous tissue formation as previously reported^61,62^.

For immunofluorescent staining, tissues were deparaffinized, and the antigens were retrieved by incubation in Proteinase K (Agilent, Carpinteria, CA) for 20 min at room temperature. Nonspecific antigens were blocked by applying Normal Donkey Serum (Jackson ImmunoResearch, West Grove, PA). Slides were stained with primary antibodies, as detailed in Supplemental Table 1. The primary antibodies were applied to the slides, after which the slides were incubated at 4 °C overnight and washed using PBS; the slides were then incubated with secondary antibodies for one hour at room temperature. Finally, the slides were stained with 4',6-diamidino-2-phenylindole dihydrochloride (DAPI, 300 nM, Invitrogen, Waltham, MA) for five minutes in the dark. ProLong™ Gold Antifade mounting medium (Invitrogen) was applied to the tissue. Images were captured using a Carl Zeiss Axio Imager Z1 fluorescent microscope (Carl Zeiss, Oberkochen, Germany) equipped with ApoTome and AxioCam HRc cameras. Negative controls were processed using identical protocols while omitting the primary antibody to exclude nonspecific staining. Images were captured with 4 × 4 tile scans at 20 × objective.

### Statistical analysis

All statistical analyses were performed using Prism 8 (GraphPad, La Jolla, CA); *p* < 0.05 was considered statistically significant. The outcome measurements were (1) BLI intensity, (2) gene expression and (3) µCT measures. Separately for each dependent measure, 2-way analysis of variance (ANOVA) or mixed-effects analysis (BLI only), was performed using mean values with the grouping of implant group; for multiple comparisons, appropriate post hoc tests were used. In the figures, median (min; max) values are shown.

### Ethics approval and consent to participate

This study was approved by the Cedars-Sinai IACUC, study #IACUC007961. All animal procedures were performed according to the ARRIVE guidelines^57^. For iPSC derivation healthy control dermal fibroblasts from one donor were obtained from the Coriell Institute for Medical Research (Camden, NJ) and additional dermal fibroblasts and blood T-cells were derived from two healthy donors at Cedars-Sinai Medical Center, an informed consent was obtained prior to cell line derivation.

## Results

### 3D printable cell-bio-ink construct maintain structure in vitro

**Figure 2:**
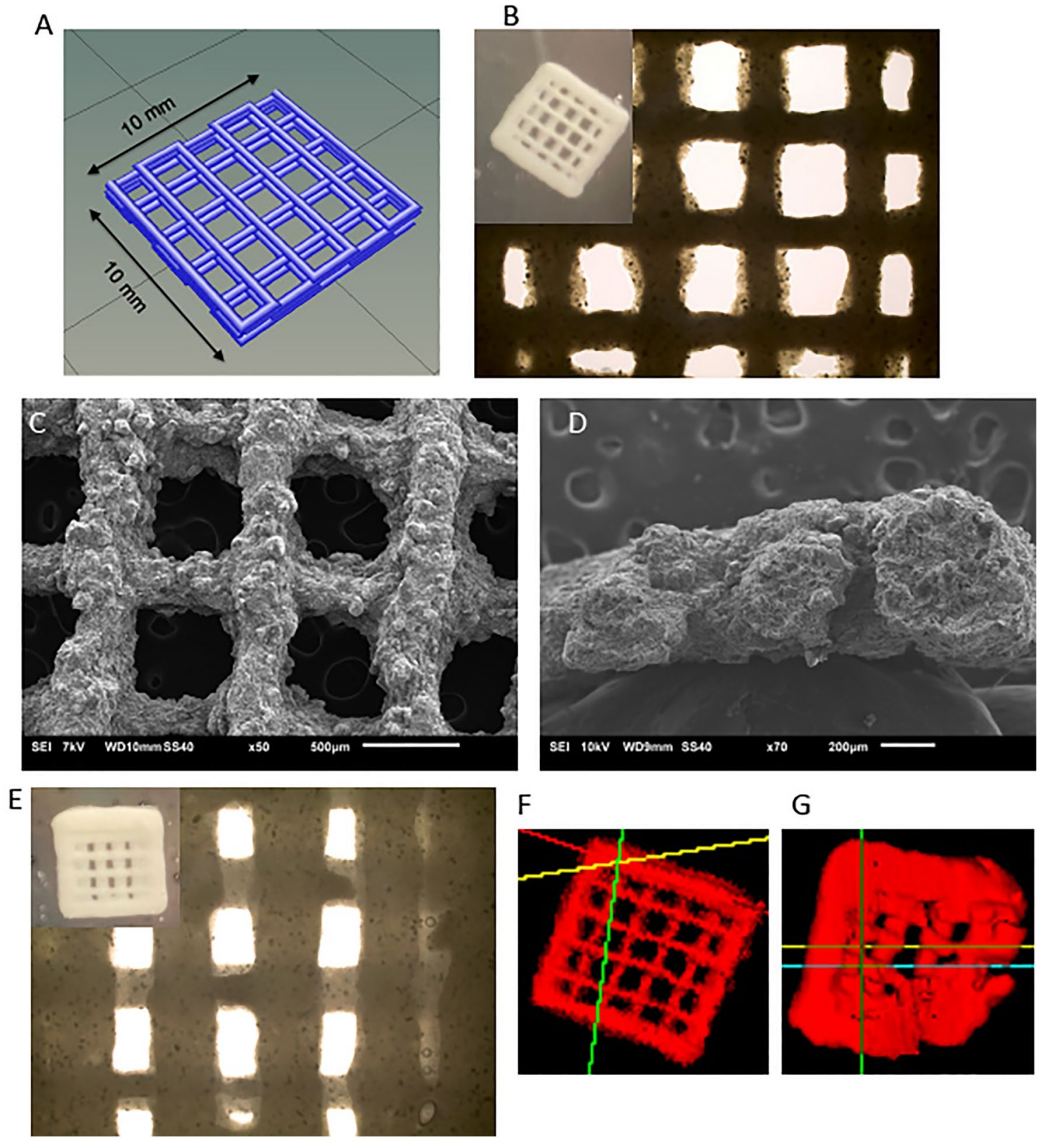
3D printed constructs for bone regeneration. 3D printed Ink-Bone construct can be designed and printed with or without cells. (**A**) Design of 3D printed construct. (**B**)–(**D**) Characterization of 3D printed constructs. (**B**) Optical image, (**C**) SEM from top view, and (**D**) SEM from cross-section of 3D printed scaffold without cells. (**E**) Optical image of 3D printed scaffold with cells (9 m/ml). The uCT images of 3D printed constructs (**F**) without cells and (**G**) with 9 m/ml cells and cultured after 27 days. In (A), the scaffold design is 10 mm × 10 mm × 2 mm square. Printed at 10 mm/s. Air pressure is 25kpa for (**B**) and (**F**), and 30kpa for (**C**)–(**E**) and (**G**). The original magnifications for (**C**) and (**D**) are ×50 and ×70 respectively.

The 3D printed construct of Ink-Bone (Fig. 2B) maintained a similar shape as our design (Fig. 2A). The edge of the construct is visibly thicker. The line grid is straight and visibly clear (Fig. 2B), and it maintains a grid pattern and clear spacing under microscopic scale (Fig. 2C). The line width is about 200–300 µm and the spacing between parallel lines is about 700–800 µm in length (Fig. 2C,D). Granular particle can be found in the SEM images (Fig. 2C,D), which are the tricalcium phosphate particles. The cross-sections of the construct are about 500 µm. The construct is thinner than the design, which could be a result of the deformation of the materials under gravity or compression. The 3D printed construct with cells (cell-Ink-Bone) construct also maintained an integral shape (Fig. 2E) as in our design (Fig. 2A). The addition of cells into the construct assumably changed the mechanical and rheological properties and thus we had to use a different printing parameter (30 kPa) to achieve a stable printing. When printing with the adjusted parameters the lines seem to be thicker and spacing to be smaller than the constructs without cells (Fig. 2B). The cell-bio-ink bone maintained a good shape at day 0 (Fig. 2F), but partly lost the integrity after 27 days culture in vitro (Fig. 2G).

### Ink-bone with both BM-MSCs and iPSC-MPCs shows increased survival and quality in vitro compared to GelXA

**Figure 3:**
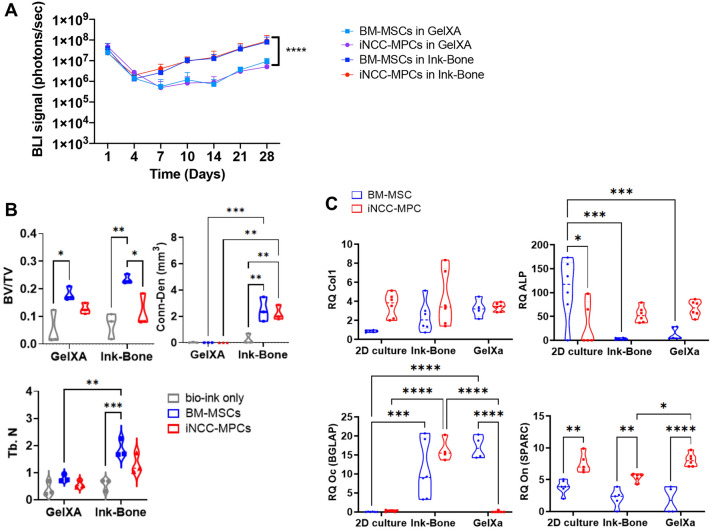
Cell viability and in vitro bone formation increased in the Ink-bone group. A. Cells were labeled with a Luciferase reporter gene and their viability in the two bio inks was assessed by BLI in vitro. *N* ≥ 4. B. Cells were cultured in 2D or in bio-ink 3D constructs in osteogenic media. Osteogenic gene expression was tested on Day 10. *N* ≥ 4. C. In vitro bone formation of cells in the bio-inks was evaluated using µCT at 3 weeks and compared to Ink-Bone only. *N* = 3. ^*^p < 0.05, ^**^p < 0.01, ^***^p < 0.001, ^****^p < 0.0001.

In the GelXa group, BLI signals were reduced between days 1 and 7. At day 14, the signal increased again. In the Ink-Bone group, the BLI signal was reduced between days 1 and 4. At day 4, the signal consistently increased until day 28. Ink-Bone resulted in significantly higher BLI signals compared to GelXA at 28 days. No differences were observed between BM-MSCs and iNCC-MPCs (Fig. 3A). In the osteogenic differentiation assay using cells mixed with both bio-inks and exposed to osteogenic media, no difference in *Col 1* levels was detected between the bio-inks and cell sources (Fig. 3B). *ALP* levels were increased in the BM-MSC group compared to iNCC-MPC. *Oc* gene expression was increased in the Ink-Bone group compared to 2D culture and GelXA in iNCC-MPCs, and in the Ink-Bone and GelXA group versus 2D culture in the BM-MSC group. In the GelXA group, BM-MSCs expressed higher levels of *Oc* compared to iNCC-MPCs. *On* was increased in the GelXA group compared to Ink-Bone. In each group, higher levels of *On* were measured in iNCC-MPCs compared to BM-MSCs (Fig. 3B).

µCT analysis in vitro demonstrated no differences in the Bone Volume/Total Volume between the bio-inks. Connectivity density was increased in BM-MSCs and iNCC-MPCs and trabecular number was increased in BM-MSCs in Ink-Bone compared to GelXa (Fig. 3C). In both bio-inks, increased BV/TV was detected in the BM-MSC group versus bio-ink only (Fig. 3C). In the Ink-Bone group, BV/TV levels were increased in the BM-MSC group compared to iNCC-MPCs. In the Ink-Bone group, increased trabecular number values were measured in the BM-MSC group compared to Ink-Bone-only controls (Fig. 3C).

### Osteogenic marker expression is increased in BMP6 transfected iNCC-MPCs and BM-MSCs mixed with ink-bone

**Figure 4:**
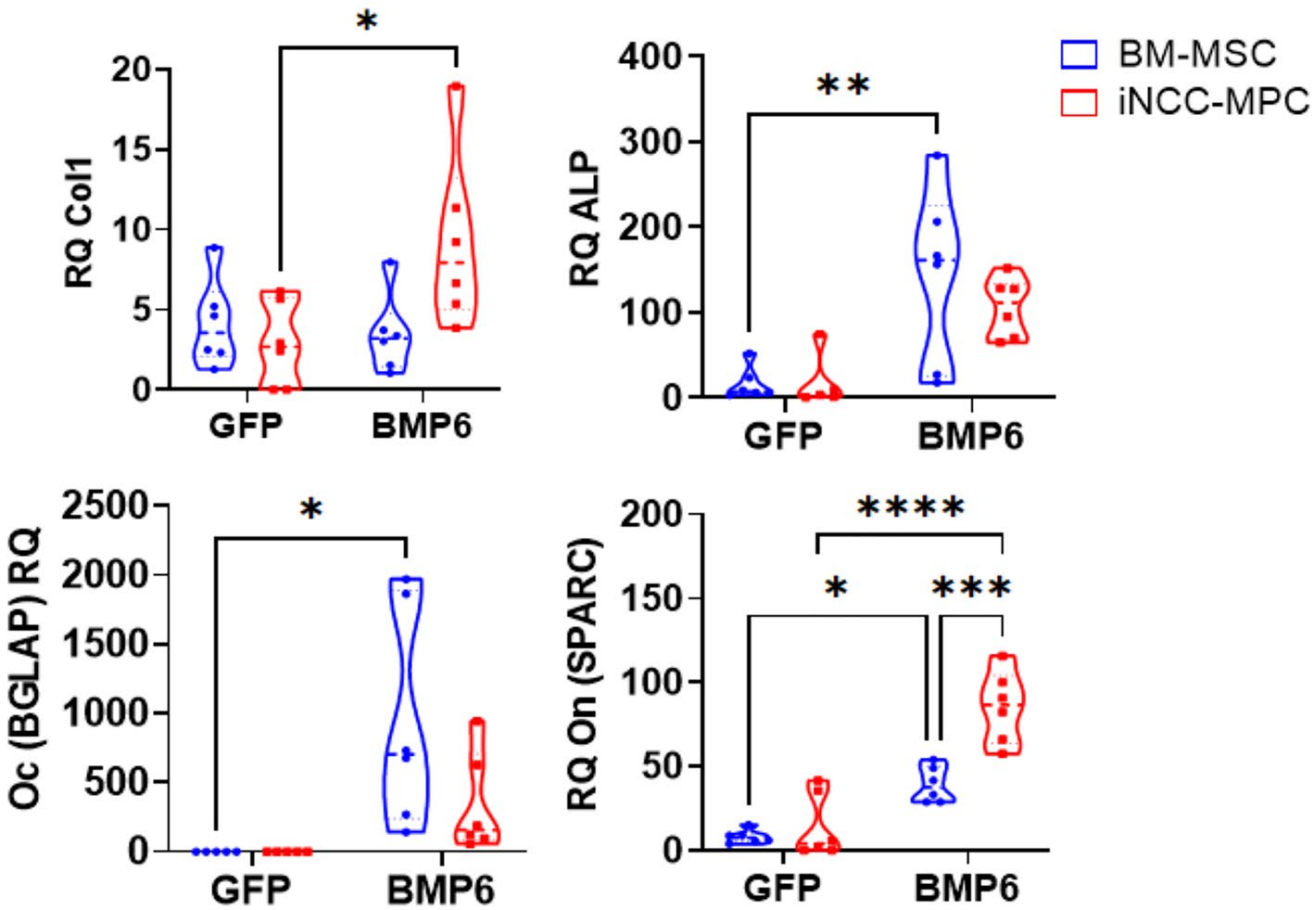
Osteogenesis marker expression increases in BMP6 transfected cells in Ink-Bone. (**A**) Cells were cultured in 2D or in bio-ink 3D constructs in osteogenic media. Osteogenic gene expression was tested on Day 10. (**B**) Gene expression of cells transfected with BMP6 and cultured in Ink-Bone constructs for 10 days. ^*^p < 0.05, ^**^p < 0.01, ^***^p < 0.001, ^****^p < 0.0001, *n* ≥ 5.

In response to BMP6 overexpression in Ink-Bone constructs, *Col I* was elevated compared to GFP controls. Comparing cell types, no statistically significant difference between cell types was detected. *ALP* and *Oc* levels were elevated in BM-MSCs in the BMP6 group versus GFP control. No differences were detected between cell types in the GFP and BMP6 groups. *On* was increased in both BM-MSCs and iNCC-MPCs in the BMP6 group versus GFP control. Within the BMP6 group *Oc* levels were elevated in the iNCC-NPC group versus BM-MSC (Fig. 4).

### Addition of Ink-Bone + iNCC-MPC-BMP6 improves frontal and parietal defect healing

**Figure 5:**
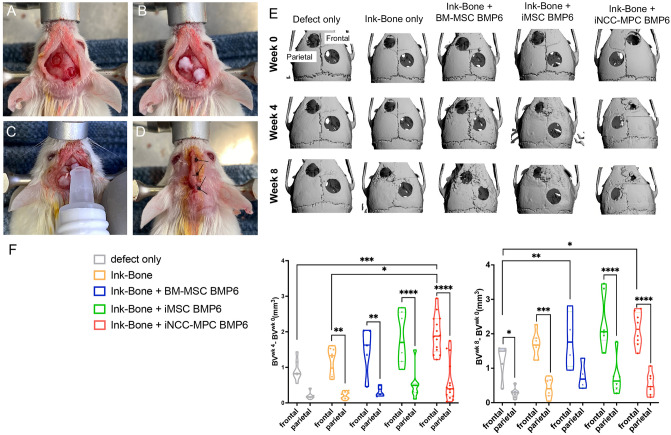
Cranial defect filling with Ink-Bone seeded with iNCC-MPC-BMP6 results in increased bone volume in the frontal bone. (**A**) Surgical approach to create calvarial defects in the frontal and parietal bone. Shown is the fixated cranium with circular defects in the frontal and parietal bone. (**B**) Filling of the defects with Ink-Bone. (**C**) Crosslinking of Ink-Bone using CaCl_2_ solution. (**D**) The sutured skin. (**E**) 3D reconstruction of the cranial defects treated with Ink-Bone with and without cells or untreated at weeks 0 and 4. (**F**) Violin plots showing the change in bone volume in the different experimental groups and implantation sites between weeks 0 and 4, and 0 and 8. *p < 0.05, **p < 0.01, ***p < 0.001, ****p < 0.0001, *n* ≥ 5.

3D µCT image reconstruction gave an overview of the optical defect sizes of the different experimental groups at week 0, 4 and 8 post-surgery, as a result of the surgical procedure (Fig. 5A–E). µCT analysis at week 4 demonstrated an enhanced relative bone volume (BV_week 4_ − BV_week 0_) in the iNCC-MPC-BMP6 group in the frontal bone compared to defect only and Ink-Bone only. At 8 weeks, the relative BV was increased in the iNCC-MPC-BMP6 as well as in the iMSC-BMP6 groups compared to defect only. No significant differences in bone volume were detected between any of the other groups in the frontal bone. Except in the BM-MSC BMP-6 group, in all frontal bone groups treated with Ink-Bone, the relative bone volume was increased compared to parietal bone (Fig. 5F).

### Increased new bone formation in frontal bone defects treated with ink-bone + iNCC-MPC-BMP6 observed with histological and immunofluorescent analyses

**Figure 6:**
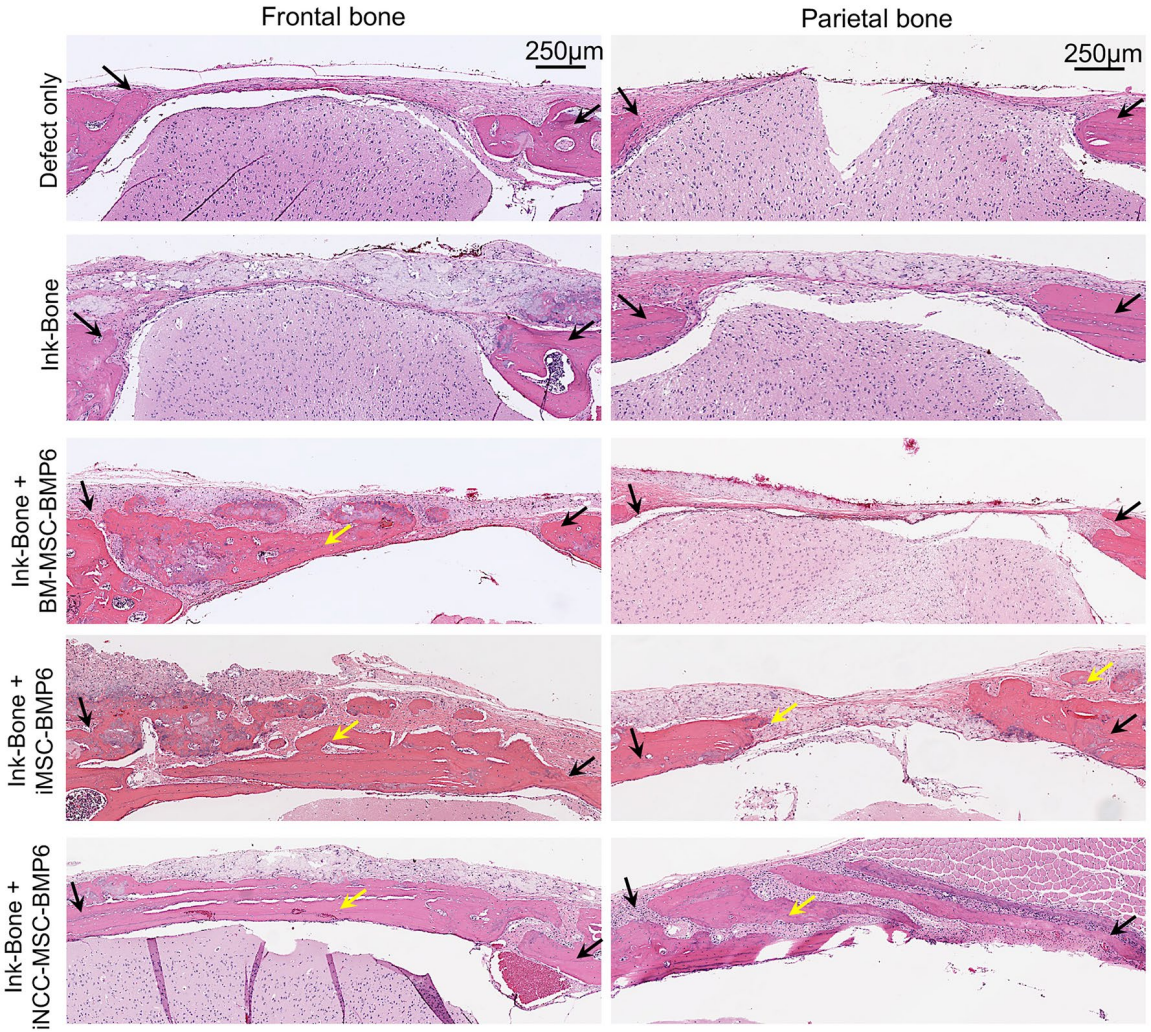
Histological evaluation at week 8 demonstrates increased new bone formation in frontal bone defects treated with Ink-Bone + iNCC-MPC-BMP6. Shown is the H&E staining of the frontal bone defects treated with the different experimental groups. Black arrows: edges of defect site, yellow arrow: new bone formation.

Histological evaluation performed at week 8 post-surgery demonstrated increased new bone formation and partial bridging in the iMSC-BMP6 and iNCC-MPC-BMP6 groups in the frontal bone. In the defect area, new bone formation was also detected in the BM-MSC group. However, no bone bridging between host bone and newly formed bone at the implantation site was detected. In the defect only and Ink-Bone only groups, no new bone formation was observed. In the parietal bone, new bone formation was detected in the iMSC-BMP6 and iNCC-MPC-BMP6 groups, but no integration of the newly formed bone into the host bone tissue was observed. In the BM-MSC, Ink-Bone only and defect only groups, no or only marginal new bone was found (Fig. 6). No remnants of Ink-Bone were found in the defects of any of the groups at the study end.

**Figure 7:**
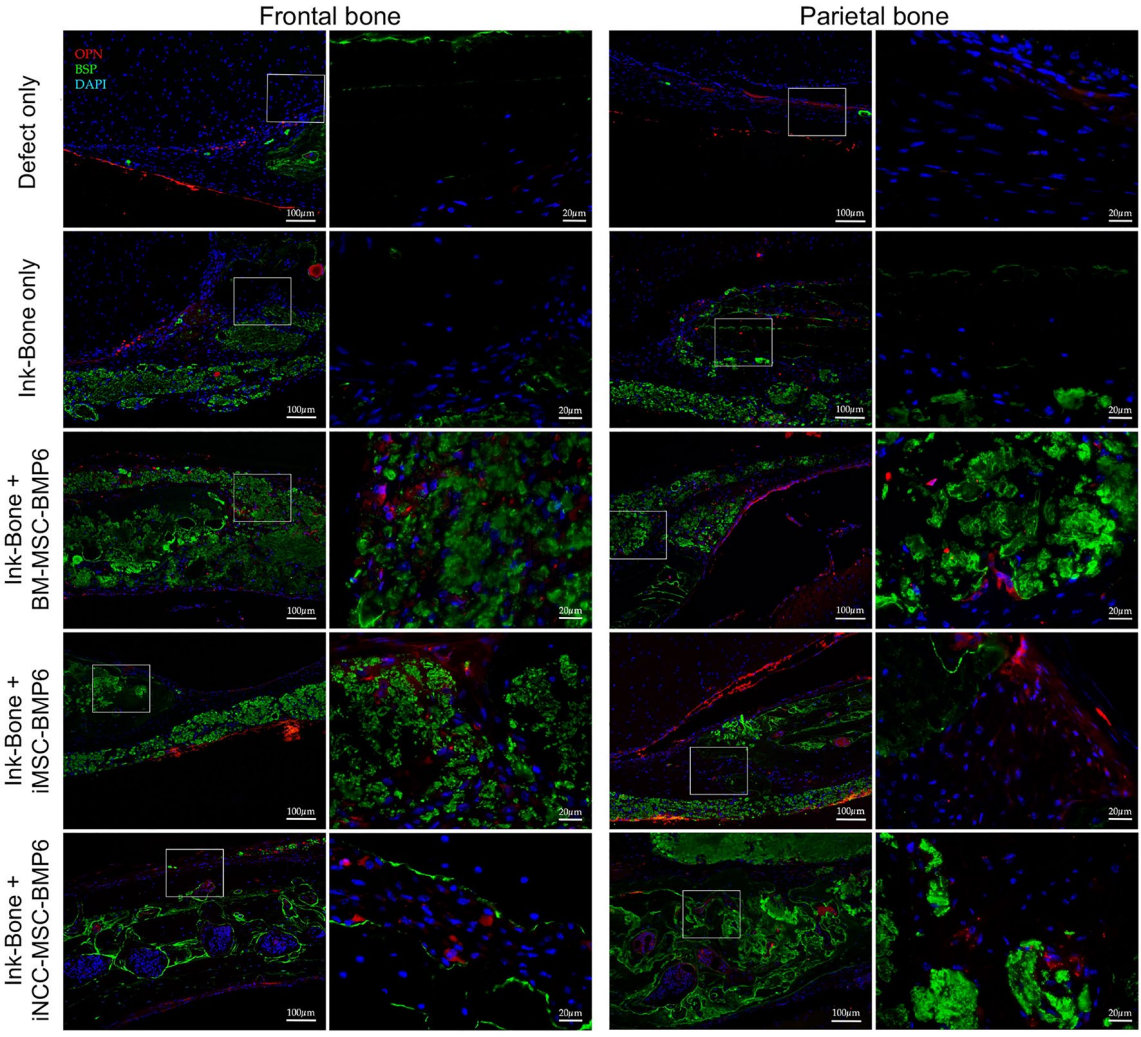
Immunofluorescence staining of the calvarial defect repair in a mouse model using Ink-Bone + iNCC-MPC-BMP6 and control groups. Immunofluorescence shows a DAPI, anti-human OPN (osteopontin) or human BSP (bone sialoprotein) staining. The antibody against BSP Scale bars indicate 100 µm in the left and 20 µm in magnified images in the right columns.

Immunofluorescent staining of the host-tissue and engineered construct (Fig. 7) at 8 weeks post implantation. Expression levels of human Bone Sialoprotein (hBSP) and Osteopontin in the cell-bio-ink treated defects are shown. Interestingly, the anti-hBSP antibody had a low unspecific staining in the defect treated with Bone-Ink only (Fig. 7).

## Discussion

The results of this study (Fig. 1) show the potential of neural crest-derived progenitor cell-incorporated bio-ink to regenerate cranial defects. We demonstrated Ink-Bone to support cell survival and to promote osteogenic differentiation of iNCC-MPCs and BM-MSCs compared to GelXA in vitro. Our in vivo data demonstrate that the combination of Ink-Bone with iNCC-MPC-BMP6 stimulates cranial defect healing in the frontal bone more efficiently than BM-MSCs and iMSCs.

BLI demonstrated that both the GelXa and the Ink-Bone bio-inks supported cell viability. No difference in cell viability depending on the cell type (BM-MSC versus iNCC-MPC) was detected. Comparing bio-inks, the luciferase signal was significantly increased at day 28 in cells embedded in Ink-Bone versus GelXa. This may be a result of blending or modification of the alginate-based bio-inks, such as Ink-Bone, since this has been shown to improve molecules adhesion^63^. Comparable with our results showing an increase in BLI signals from day 4 on in the nanocellulose-alginate based Ink-Bone, an increase in cell viability between days 0–7 was demonstrated in a study using chondrocyte-incorporated nanocellulose-alginate bio-ink^64^.

In the presence of osteogenic media, osteocalcin levels increased in BM-MSCs embedded in Ink-Bone and osteonectin in iNCC-MPCs embedded in GelXa, extracellular bone matrix proteins that have been shown to have synergistic effects in MSC proliferation and osteogenic differentiation^65^. The expression of osteogenic differentiation markers in our study is in line with prior studies showing MSC differentiation in alginate-based bio-inks^13^.

µCT analysis of in vitro cultured bio-inks with and without cells resulted in increased BV/TV and trabecular number in the BM-MSC group and increased connectivity density in both the BM-MSC and iNCC-MPC groups compared to bio-ink only. Connectivity density and trabecular numbers were increased in the Ink-Bone group compared to GelXa group in the presence of MSCs. µCT has been used since it provides a powerful platform to analyze, visualize, and explore the bio-ink scaffolds in a 3D fashion^66^. The observed increased bone formation of bio-inks combined with BM-MSCs is likely due to the strong intrinsic osteogenic potential of BM-MSCs. Multiple studies suggest that BM-MSCs are strongly involved in the process of heterotopic ossification, a process of ectopic bone tissue formation in non-bone tissues besides the potential effect of MSCs on tissue regeneration^67^. In addition to the capability of new bone formation, bone quality is an important factor when considering a bio-ink for cranial repair. For example, in prior studies it has been shown that relatively smaller pores and a larger specific surface area assist cell attachment^68^. The increased bone volume and connectivity values in the Ink-Bone group indicate that this biomaterial is more suitable for MSC function and was, therefore, chosen for subsequent in vivo studies comparing the different cell types.

BMP6 transfection of BM-MSCs and iNCC-MPCs embedded in Ink-Bone resulted in an increase of the known osteogenic markers, Col1, ALP, Oc and/or On. These findings confirm the strong osteogenic potential of BMP6 that has been demonstrated by our group and others^35–37^. The osteogenic marker expression between BMP6-transfected BM-MSCs and iNCC-MPCs embedded into Ink-Bone differed depending on the marker gene. However, both cell types responded to the BMP6 overexpression with osteogenic marker expression.

Our μCT data and histological analysis of the bone defects demonstrate an improved relative bone volume in frontal bone defects that were treated iNCC-MPC-BMP6 cell containing Ink-Bone at 4 weeks post-surgery. At 8 weeks, the relative BV was increased in the iNCC-MPC-BMP6 and iMSC-BMP6 groups compared to defect only, but not in the BM-MSC-BMP6 group. In a recent study by our group, the BM-MSC-Luc2-seeded allograft group showed increased BV compared to allograft only and to iNCC-MPC-Luc2-seeded allograft groups at 8 weeks post-surgery using the same mouse strain^56^. The difference in findings is likely due to several reasons: In the current study, 2 mm defects in distinct areas of the frontal and parietal cranium were created, while we created 5 mm defects that included part of the lambdoid and sagittal cranial sutures in the previous study. Furthermore, we transfected the cells with BMP6 in the present study and embedded the cells in Ink-Bone. Similarly, other prior studies showing an improvement of cranial defect repair using BM-MSCs applied with and without carriers created larger defects of at least 5 mm in rats that included cranial sutures^69,70^. Therefore, it is possible that stem cells present in the sutures^71^, contribute to bone regeneration in response to BM-MSC therapy in studies with larger cranial defects.

Similar to our study, Kuhn et al. demonstrated an increased bone regenerative potential of induced human embryonic stem cells on calcium phosphate cement scaffolds versus BM-MSCs in the cranium^72^. In contrast, implantation of osteo-induced iPSCs and BM-MSCs seeded on biofunctionalized macroporous calcium phosphate cement showed a similar quality of new bone during cranial regeneration^73^.

Interestingly, no increase in bone regeneration was detected in the parietal bone groups that are developmentally derived from the mesoderm. Based on our findings other strategies should be considered when treating parietal bone defects. A potential treatment source may be stem cells from the cranial sutures, as these cells have shown to have great healing potential^71^.

Immunofluorescent staining of the cell-bio-ink constructs (Fig. 7) indicated survival of the implanted cells for at least 8 weeks. Expression of human Bone Sialoprotein (hBSP) and Osteopontin in the cell-bio-ink treated defects indicates differentiation of the cells to osteoblasts, new bone tissue formation and defect repair by the implanted cells. Alternatively or in addition to the observed effects, it is possible that factors secreted by the implanted cells or that BMP-6 stimulated host cell recruitment to the implantation site, leading to hBPS and OPN expression.

This study is not without limitations: Due to the animal model we have chosen, the defect size we were able to create was only 2 mm in diameter. While this defect was clearly large enough in size to show no bone healing in the control group (defect only), this defect size is too small to allow for the addition of 3D printed bio-ink. Larger defects in larger animal models, such as in pigs, are needed to allow for application of 3D printed cell containing Ink-Bone. Cranial bone healing is known to be challenging, especially in areas without cranial sutures. While our study clearly showed that the combination of bio-printable bio-ink in combination with BMP6 transfected iNCC-MPCs is capable of stimulating bone regeneration, we still did not achieve full bone bridging, especially in the parietal bone. Bio-printing of the therapeutic candidate may help to overcome these challenges since nano-topography of materials have been shown to be important for osteogenic differentiation^74^. Additionally, the Ink-Bone crosslinking might be suboptimal. Newer technologies to crosslink 3D printable biomaterials should be tried in the future to improve the mechanical properties of the construct, for example vat polymerization-based^75^.

Furthermore, we did not detect significant differences between the Ink-Bone + BM-MSC, Ink-Bone + iMSC and Ink-Bone + iNCC-MPC groups. The reasons may be the large standard deviations that we detected. These may be due to differences in host response to the treatment or BMP6 expression levels of the different cell batches. Also, use of more osteoconductive biomaterials could possibly result in better bone formation”.

## Summary and conclusion

The results of this study show the potential of stromal cell-incorporated bio-ink to stimulate regeneration in frontal cranial defects. We demonstrated Ink-Bone to be beneficial for the survival and osteogenic differentiation of iNCC-MPCs and BM-MSCs compared to GelXA in vitro. The printability of bio-ink scaffolds and their ability to support stromal cell survival and osteogenic differentiation makes them attractive for craniofacial reconstruction. Our in vivo data demonstrate that the combination of Ink-Bone with BMP6 overexpressing iNCC-MPCs stimulates cranial defect healing in the frontal bone more efficiently than BMP6 overexpressing BM-MSCs and iMSCs. Employment of large animal models with cranial defects of 20 mm minimum will allow for testing of 3D printed stromal cell-embedded woven bio-ink, which may further stimulate graft-host bone integration.

## Supplementary Information

Supplementary Table S1.

## Data Availability

The dataset(s) supporting the conclusions of this article is(are) included within the article [and its additional file(s)].

## Abbreviations

BM-MSC: Bone marrow mesenchymal stromal cells
rhBMP: Recombinant human bone morphogenetic protein
NC: Neural crest
NCC: Neural crest cell
iPSC: Induced pluripotent stem cell
iNCC-MPC: IPSC-derived NC mesenchymal progenitor cells
Ink-Bone: CELLINK BONE
CelXA: GelXA BONE
BLI: Bioluminescent imaging
Luc2: Luciferase2
ROI: Region of interest
Oc: Osteocalcin
Col I: Collagen type I
ALP: Alkaline phosphatase
ON: Osteonectin
OPN: Osteopontin
SQ: Subcutaneous
BV: Bone volume
TV: Total volume
H&E: Hematoxylin and eosin
DAPI: 4',6-Diamidino-2-phenylindole dihydrochloride
hBSP: Human bone sialoprotein
