# Supplementary material for: iPSC-neural crest derived cells embedded in 3D printable bio-ink promote cranial bone defect repair
Source: Sci Rep. 2022 Nov 4;12:18701. doi: 10.1038/s41598-022-22502-8 (PMC9636385; doi:10.1038/s41598-022-22502-8)
Supplement: Supplementary file 1 — Supplementary Table S1. [file 41598_2022_22502_MOESM1_ESM.docx]

Supplement Table 1:

| Type | Antigen | Conjugation | Host | Reactivity | Company | Catalog # | Dilution |
| --- | --- | --- | --- | --- | --- | --- | --- |
| 1 | Chicken Polyclonal Bone Sialoprotein | - | Chicken | Human | US Biologicals | S1013-34M | 1:40 |
| 1 | Goat Polyclonal Human Osteopontin | - | Goat | Human | R&D Systems | AF1433 | 1:166.7 |
| 2 | IgG (H+L) | Cy5 | Donkey | Goat | Jackson ImmunoResearch | 705-606-147 | 1:800 |
| 2 | IgY (IgG)　(H+L) | Cy3 | Donkey | Chicken | Jackson ImmunoResearch | 703-166-155 | 1:800 |
